# Computational drug discovery approaches identify mebendazole as a candidate treatment for autosomal dominant polycystic kidney disease

**DOI:** 10.3389/fphar.2024.1397864

**Published:** 2024-05-23

**Authors:** Philip W. Brownjohn, Azedine Zoufir, Daniel J. O’Donovan, Saatviga Sudhahar, Alexander Syme, Rosemary Huckvale, John R. Porter, Hester Bange, Jane Brennan, Neil T. Thompson

**Affiliations:** ^1^ Healx Ltd., Cambridge, United Kingdom; ^2^ Crown Bioscience Netherlands B.V., Biopartner Center Leiden JH, Leiden, Netherlands

**Keywords:** autosomal dominant polycystic kidney disease, rare diseases, gene expression profiling, machine learning, drug discovery, drug repositioning, mebendazole, tubulin modulators

## Abstract

Autosomal dominant polycystic kidney disease (ADPKD) is a rare genetic disorder characterised by numerous renal cysts, the progressive expansion of which can impact kidney function and lead eventually to renal failure. Tolvaptan is the only disease-modifying drug approved for the treatment of ADPKD, however its poor side effect and safety profile necessitates the need for the development of new therapeutics in this area. Using a combination of transcriptomic and machine learning computational drug discovery tools, we predicted that a number of existing drugs could have utility in the treatment of ADPKD, and subsequently validated several of these drug predictions in established models of disease. We determined that the anthelmintic mebendazole was a potent anti-cystic agent in human cellular and *in vivo* models of ADPKD, and is likely acting through the inhibition of microtubule polymerisation and protein kinase activity. These findings demonstrate the utility of combining computational approaches to identify and understand potential new treatments for traditionally underserved rare diseases.

## Introduction

A member of the wider group of ciliopathic disorders, autosomal dominant polycystic kidney disease (ADPKD) is caused primarily by mutations in the PKD1 or PKD2 genes, encoding the ciliary proteins polycystin 1 (PC1) and polycystin 2 (PC2), respectively (Bergmann et al., 2018). Pathophysiologically, the disease is characterised by the formation and expansion of fluid-filled cysts throughout the parenchyma of the kidney, and in some cases extra-renal organs such as the liver and pancreas. The variable but progressive growth of kidney cysts leads to structural remodelling of the kidney and eventually a significant impairment in renal function in most cases, with a median age of progression to end-stage renal disease (ESRD) of 58 years (Spithoven et al., 2014a). While ADPKD is classified as a rare disease with a prevalence of 3-5 per 10,000 (Willey et al., 2016; Solazzo et al., 2018; Willey et al., 2019), it is the most common genetic cause of renal failure, accounting for approximately 10% of all patients requiring renal replacement therapy (Spithoven et al., 2014b), and remains a significant public health burden. Tolvaptan is the only approved therapy targeting the underlying pathophysiology of ADPKD, and has demonstrated moderate efficacy in slowing renal cyst growth and preserving kidney function (Torres et al., 2012; Torres et al., 2017a; Torres et al., 2017b). However, the common tolvaptan side effect of polyuria and the rare but serious complication of idiosyncratic hepatic injury mean there remains an unmet need for safer and more effective therapies for ADPKD patients.

PC1 and PC2 are transmembrane proteins which form a multimeric complex in a number of cellular membranes, including the primary cilia, where they are thought to transduce extracellular signals through the regulation of a plethora of intracellular pathways. Disease-causing mutations in either gene product result in dysregulation of these intracellular signalling cascades, most notably in kidney epithelial cells, with a reduction in intracellular Ca^2+^ and elevation of cAMP leading to aberrant modulation of downstream pathways converging on defects in cell polarity, and increases in cell proliferation and extracellular fluid secretion (Bergmann et al., 2018; Gall et al., 2019). Beyond primary processes driving cyst initiation and expansion, secondary interstitial inflammation and fibrosis have been implicated in later stage ADPKD pathology and ESRD (Song et al., 2017; Zhang et al., 2020). As the mechanistic understanding of key pathogenic pathways has evolved, so too have opportunities for therapeutic intervention. Tolvaptan is an antagonist of the vasopressin type-2 receptor (V2R), and negatively impacts cyst growth through the repression of aberrantly elevated cAMP levels in collecting duct cells (Reif et al., 2011). In line with this primary mechanism of action however, tolvaptan also induces significant clinical aquaresis, which can present a barrier to initiating or maintaining treatment, particularly in younger patients with preserved estimated glomerular filtration rate who would most benefit from early and prolonged intervention (Müller et al., 2021; Calvaruso et al., 2023). While the mechanism is not well understood, there also remains a small but significant risk of tolvaptan-induced hepatic injury, which necessitates frequent and regular monitoring of liver function for the duration of treatment and further increases the burden on patients and healthcare providers (Müller et al., 2021). Beyond V2R antagonism, a number of diverse mechanisms for therapeutic intervention have been evaluated in randomised controlled clinical trials, including mTOR inhibition (Kim and Edelstein, 2012), tyrosine kinase inhibition (Tesar et al., 2017), AMP-activated protein kinase (AMPK) activation (Perrone et al., 2021; Brosnahan et al., 2022) and somatostatin receptor agonism (Griffiths et al., 2020). Unfortunately these efforts have yet to yield any further regulatory approvals, and thus there remains a significant need to identify additional tractable targets and mechanisms for the treatment of ADPKD.

Drug discovery for rare diseases suffers from a unique set of challenges, not least of which is an often incomplete understanding of disease pathogenesis from which to identify new therapeutic targets. Despite advances in unravelling the molecular aetiology of ADPKD, the complex processes involved give rise to a plethora of putative targets for prioritisation, validation and development. In order to address this problem using a target agnostic approach, we applied computational drug discovery approaches to interrogate publicly available gene expression datasets and an ADPKD-augmented rare disease knowledge graph to identify and prioritise clinical-stage candidate drugs for the treatment of ADPKD. We screened a focused library of drug predictions through preclinical disease model systems, and validated the antiparasitic drug mebendazole as an effective treatment in human cellular models and a genetic animal model of ADPKD. Using *in silico* and experimental approaches, we further probed the putative mechanisms of action of mebendazole in ADPKD. This work highlights the utility of combining computational and experimental methods in the discovery of new treatments and therapeutic targets for rare diseases such as ADPKD, which often suffer from complex or incompletely understood pathophysiological processes.

## Materials and methods

### Gene expression datasets and differential expression analysis

Gene expression files were downloaded from GEO under accession numbers GSE7869 (Song et al., 2009), GSE24352 (Pandey et al., 2011) and GSE72554 (Menezes et al., 2016). These represented the highest quality datasets available to model ADPKD gene expression at the time this study was conducted, representing both mouse and human PKD1-driven ADPKD signatures across different stages of kidney cyst development.

Differential expression analyses were carried out on these datasets using QIAGEN Omicsoft Suite to create disease stage-specific gene signatures, a summary of which can be seen in Table 1 below. Following robust multi-array average normalisation (Irizarry et al., 2003), differential expression was performed with Limma (Ritchie et al., 2015), and *p*-values were re-adjusted with the Benjamini-Hochberg correction for multiple testing (Benjamini and Hochberg, 1995). Disease vs. normal gene signatures were generated by comparing differentially expressed genes from cystic kidney tissue to healthy kidney tissue. We used a human dataset to compare cystic tissue isolated from ADPKD patients to healthy renal cortical tissue from nephrectomised kidneys (Song et al., 2009), and used mouse datasets to compare cystic kidneys isolated from early-onset (MGI:2182840) (Pandey et al., 2011) and late-onset (MGI:3612341) (Menezes et al., 2016) mouse models of ADPKD to healthy kidneys isolated from control littermates. In the late-onset mouse model dataset, disease vs. normal gene expression signatures were further segregated by sex, as this characteristic was determined to be the largest secondary source of variation in gene expression by the original authors. ADPKD progression signatures were generated by comparing differentially expressed genes from late cystic tissue to early or minimally cystic tissue. We used the human dataset to compare cystic kidney tissue isolated from ADPKD patients to minimally cystic kidney tissue from the same patients, and used the early-onset mouse model dataset to compare cystic tissue from kidneys isolated late in the disease stage (E17.5) to cystic tissue from kidneys isolated earlier in the disease stage (E14.5).

**TABLE 1:** Summary of ADPKD gene expression data.

GEO dataset	Species	Model	Disease signature (contrast) description			
			ID	Type	Cystic stage	Sex
GSE7869 Song et al. (2009)	Human	Patient-derived cystic kidney tissue. Cysts of different sizes + minimally cystic samples. Control normal tissue from nephrectomised kidneys	175	Disease vs. Normal	Late + Early	Unknown
			176	Progression	Late vs. Early	Unknown
GSE24352 Pandey et al. (2011)	Mouse	*Pkd1-null* (MGI:2182840) embryonic kidneys. Model rapidly develops progressive kidney cysts from embryonic day (E) 15.5d	203416	Disease vs. Normal	E17.5	Unknown
			203343	Progression	E17.5 vs. E14.5	Unknown
GSE72554 Menezes et al. (2016)	Mouse	*Pkd1-conditional null* (MGI:3612341) adult kidneys. Later-onset model with *Pkd1* conditionally deleted at postnatal day (PND) 40	203348	Disease vs. Normal	PND102-210	Female
			203349	Disease vs. Normal	PND102-210	Male

### Disease gene expression matching

A variation of connectivity mapping (Lamb et al., 2006) was applied to match ADPKD disease signatures to potential drug treatments. Briefly, drug signatures derived from cell line treated expression profiles were downloaded from clue.io and processed according to the supplementary methods file from the connectivity mapping study (Lamb et al., 2006). The subsequently generated drug signatures were matched against the top 500 significantly upregulated and downregulated genes from Disease vs. Normal signatures and Progression signatures (c.f.Table 1) using the connectivity score. The connectivity score is an enrichment metric based on the Kolmogorov-Smirnov statistic to measure how closely the gene expression signature of a disease aligns with the gene expression patterns found in a set of drug signatures. It does so by examining where the up- and down-regulated genes from the disease are located in a ranked list of genes representing drug treatment effects on cell gene expression. In these drug signatures, the most relevant genes are typically found at the very top or bottom of the ranked list. A higher connectivity score indicates that the up- and down-regulated genes from the disease are concentrated in these high-priority areas, suggesting a stronger alignment between disease and drug, and a possible therapeutic connection. A caveat of the original connectivity mapping approach is that it only considers one way to represent the input disease signature, i.e., systematically considering an arbitrary number of up- and downregulated genes. However, sometimes, downregulated genes drive the disease phenotype more than upregulated genes and *vice versa*. To that end, we developed disease gene expression matching (DGEM) which incorporates the original connectivity mapping method, as well as an additional module that extracts an optimal configuration for the input disease signature, i.e., the configuration which yields the higher connectivity score for a set of control drug signatures. This set of control drug signatures is selected to represent an ideal therapeutic gene expression profile to be matched against the input disease signature. Note that the standard connectivity mapping routine is not modified, only the disease input signature is adjusted to better model the disease expression according to the target therapeutic control signature.

In this study, two configurations were investigated: 1. The top 250 up- and 250 downregulated genes and 2. The top 500 differentially expressed genes regardless of direction. Then for each disease contrast (c.f. Table 1), the optimal configuration was obtained by running connectivity mapping and assessing which configuration produced the highest connectivity score for the control drug(s). In this case, metformin was selected as a sole control treatment from which to benchmark, as it had at the time demonstrated clinical efficacy against ADPKD relevant functional endpoints (Pisani et al., 2018), and importantly, was represented within the CMap library, unlike other potential clinical controls such as tolvaptan. For each disease signature, drug predictions were ranked in descending order of the highest absolute connectivity score. All factors related to a drug treatment (such as concentration and cell line) were condensed into a single rank for each drug. This was done by taking the highest connectivity score from all the different signatures that represent these experimental conditions for that drug.

### Consensus pathway enrichment analysis

To enrich the annotation of drug-disease gene expression signatures with putative pathway terms, distinct gene sets representing the therapeutic action of mebendazole and cloperastine in ADPKD disease (mebendazole-ADPKD, cloperastine-ADPKD respectively) were compiled. These sets were derived from the principal genes — specifically, the top 100 overlapping genes irrespective of directional influence — underpinning the connectivity scores between each drug signature and its matching disease signature (c.f. Table 1 and Supplementary Table S1). Furthermore, signatures obtained from multiple concentrations of each drug were amalgamated to formulate a unified signature for each drug. Overlapping genes were only considered from signatures with a connectivity score ≥0.5. Consensus pathway enrichments (WikiPathways 2019) were obtained using the Enrichr Consensus Terms Appyter (https://appyters.maayanlab.cloud/#/Enrichr_Consensus_Terms) (Wang et al., 2013; Wang et al., 2016). WikiPathways was chosen as the main pathway database because it has comprehensive information on rare disease pathways. Its strong curation process, which includes automatic quality checks and a clear review system, ensures that only verified and approved content is included (Martens et al., 2020).

### ADPKD-enriched knowledge graph

Treatment predictions were made from an ADPKD enriched knowledge graph using an algorithm derived from a method published by Himmelstein et al., 2017. The graph consists of 165 K nodes of 8 different types (DRUG, DISEASE, GENE, etc.) associated by over 700 M connections sourced from published data sets and internally curated Healx data. The algorithm calculates features for the disease using a “degree weighted path count” metric from over 450B paths. This data set is then used to train a neural network to recognise features indicative of known treatments for similar diseases. The trained network was then used to suggest novel treatments from a set of 20 K drugs connected to the disease in the graph. When tested using a held out set of known treatments, the algorithm has produced an AUC score of 0.906 (+/− 0.12) and F1 score of 0.894 (+/− 0.16).

### Multi-scale interactome

The multi-scale interactome (MSI) algorithm (Ruiz et al., 2021) plays a crucial role in elucidating potential treatments for ADPKD by comprehensively analysing the intricate network of molecular interactions within cells as defined in a knowledge graph. By integrating graph data across various biological scales, from molecular to cellular levels, the algorithm learns diffusion profiles which encodes the effects of drugs and diseases propagating through proteins and biological functions.

The knowledge graph used includes many edges including disease-protein (72K edges), drug-protein (52K edges), protein-protein (300K edges), protein-biological functions (44K edges), and biological function-biological function (63K edges). The MSI algorithm, through diffusion profiles, was used to encode the propagation effects for every disease and drug through proteins and biological functions in the knowledge graph.

The diffusion profiles are encoded through biased random walks that start at the drug or disease graph node. These profiles depend on scalar weights $w_{drug},w_{disease},w_{protein},w_{biological\;function}$ , and the probability α of continuing the walk. In this study, we used the default parameters determined by Ruiz et al., 2021: $w_{drug}=3.21,w_{disease}=3.54,w_{protein}=4.40,w_{biological\;function}=6.58$ and α=0.859 . By leveraging these encodings, the most relevant proteins and biological functions in the drug and disease diffusion profiles were ranked. For the predicted ADPKD treatments, we extracted paths between the drug and the disease node ‘ADPKD’ in the knowledge graph, filtering those paths that involve only the top k (here, k = 10) ranked proteins and biological functions. This process generated subgraphs (depicted in Figures 4A,B) that facilitate the identification of potential therapeutic targets and the exploration of treatment strategies tailored to the unique molecular dysregulations associated with ADPKD.

### Cell culture

*In vitro* studies in patient-derived 3D kidney cyst cultures were conducted by OcellO B.V. (Leiden, the Netherlands), since acquired by Crown Bioscience. Patient genotypes were as follows: Donor 1 (PKD1: c.10594C>T Gln3532*), Donor 2 (PKD1: c.5622G>A p.Trp 1874*) & Donor 3 (PKD1: c.5861dup p. (Asn1954Lysfs*36)). Primary ADPKD patient kidney cells derived from resected patient kidneys were mixed with PrimCyst-Gel (Crown Bioscience B.V.) and cultured in 384-well plates with minimally supplemented kidney base medium (Crown Bioscience B.V.) for 24 h, after which the cells were treated with or without 2.5 µM of the cyst swelling stimulus 1-deamino-8-D-arginine vasopressin (ddAVP) and varying concentrations of tolvaptan or test compound. Treatment with staurosporine at 250 nM was used as a positive control for cell death. After 48 h, cultures were fixed, permeabilised and stained with rhodamine-phalloidin and Hoechst before imaging. Cysts were segmented using detection of Hoechst-stained nuclei and Rhodamine-phalloidin-stained cellular f-actin, and cyst area determined by calculating the area in pixels of each object in every in-focus plain, which was then averaged per well (Ominer^®^ image analysis software, Crown Bioscience B.V.). Cyst area was normalised to DMSO-treated control wells (0%), and in stimulated conditions additionally to ddAVP-treated control wells (100%).

### Kinase screen

Kinase screening data was generated by the KinaseProfiler service at Eurofins Discovery (Le Bois l’Evêque, France). Using a radiometric assay system, mebendazole (10 µM) was screened against a panel of 94 human kinase targets using the Km ATP concentration for each kinase. Percentage inhibition was reported relative to uninhibited control conditions.

### Animal studies

*In vivo* work was conducted at InnoSer Belgie N.V (Diepenbeek, Belgium), according to standard operating procedures and methods described by the Association for Assessment and Accreditation of Laboratory Animal Care, and utilised the tamoxifen-inducible iKsp-*Pkd1* ^del^ mouse as previously described (Leeuwen et al., 2007; Leonhard et al., 2016). Animals were housed in individually ventilated cages with sterilised corn cob bedding at 21°C ± 2°C and 40%–70% humidity on a 12/12 dark/light cycle with *ad libitum* access to food and water.

Kidney-specific disruption of the Pkd1 gene was induced in KspCad-CreER^T2^;*Pkd1* ^lox,lox^ mice by oral tamoxifen administration (150 mg/kg/day PO) on PND18, 19 & 20, and compound administration began at PND42 and continued until study termination on PND110. Mebendazole was solubilised in 10% DMSO: 90% 2-Hydroxypropyl-b-cyclodextrin (20% w/v in physiological saline), and administered PO at 10, 20 or 30 mg/kg QD, or 10 or 15 mg/kg BID (BID 5/7 days, QD 2/7 days), and was compared with a matched QD vehicle treated group. The vasopressin V2 receptor antagonist tolvaptan was dosed in medicated food at 0.1% w/w (prepared by SSNIFF, Germany) as a positive control. Tamoxifen untreated mice, in which the Pkd1 locus remained intact (Pkd1 WT) served as a negative control. Blood urea was measured weekly from PND74 in 50–100 µL of blood sampled from the submandibular vein, and once blood urea levels reached 20 mmol/L, ESRD was said to have developed, and animals were sacrificed. The study was terminated when at least 50% of animals in the P18 iKsp-*Pkd1* ^del^ vehicle-treated group developed ESRD, which occurred in this study at PND109. One hour after final dosing on PND110, all remaining study animals were sacrificed by exsanguination via cardiac puncture under isoflurane anaesthesia, followed by cervical dislocation. Blood was processed for measurement of terminal blood urea, and both kidneys were removed from the abdominal cavity, weighed, and processed for histopathology as detailed below. Animals found dead or euthanised for reasons other than ESRD are detailed in Supplementary Figure S1, and were excluded from statistical analysis.

### Histopathology

Kidneys were fixed in 10% formalin for 24 h, after which they were cut in the transverse direction and stored in 70% ethanol. After paraffin embedding, kidneys were sectioned, stained with hematoxylin and eosin and digitally scanned for assessment of cystic load. For evaluation of cystic index, a colour thresholding method was applied using the image analysis system HALO (Indica Labs, Albuquerque, NM, United States) to identify total cystic area of each section (sum of all lesions with a lumen diameter > 9 µm), which was then normalised to total section area (excluding dilated veins and the pelvic cavity) using the following calculation: (cystic area/total area) x 100%. For the semi-quantitative evaluation of cystic grade, kidney sections were scored on an ordinal scale of 0–5, with 0.5 intervals, based on the following criteria: 0 = no cysts visible; 1 = from 1 to a few, scattered small cysts; 2 = mild number of cysts; 3 = moderate number of cysts; 4 = numerous cysts; 5 = almost all of the parenchyma replaced by cysts.

### Ultrasound

Mice were anaesthetised with isoflurane, and hair removed from the right side of the abdomen prior to collection of a 3D ultrasound scan of the complete right kidney with a Vevo 3100 imaging system (FUJIFILM VisualSonics Inc., Toronto, Canada).

### Statistical analysis

Statistical analysis and curve fitting was performed using GraphPad Prism 9.5.1. For *in vivo* study data, normality was first evaluated using D’Agostino-Pearson’s omnibus K2 test. If data were normally distributed, or could be corrected using log transformation, parametric analysis was performed using one-way ANOVA prior to Dunnet’s multiple comparisons test. If data were ordinal or not normally distributed, non-parametric analysis was performed using the Kruskal-Wallis test prior to Dunn’s multiple comparisons test. The Log-rank (Mantel-Cox) test was used for kidney survival analysis. Statistical significance for all testing was assumed when *p* < 0.05. Data are presented as mean ± standard deviation for all *in vitro* and *in vivo* data.

## Results

**FIGURE 1:**
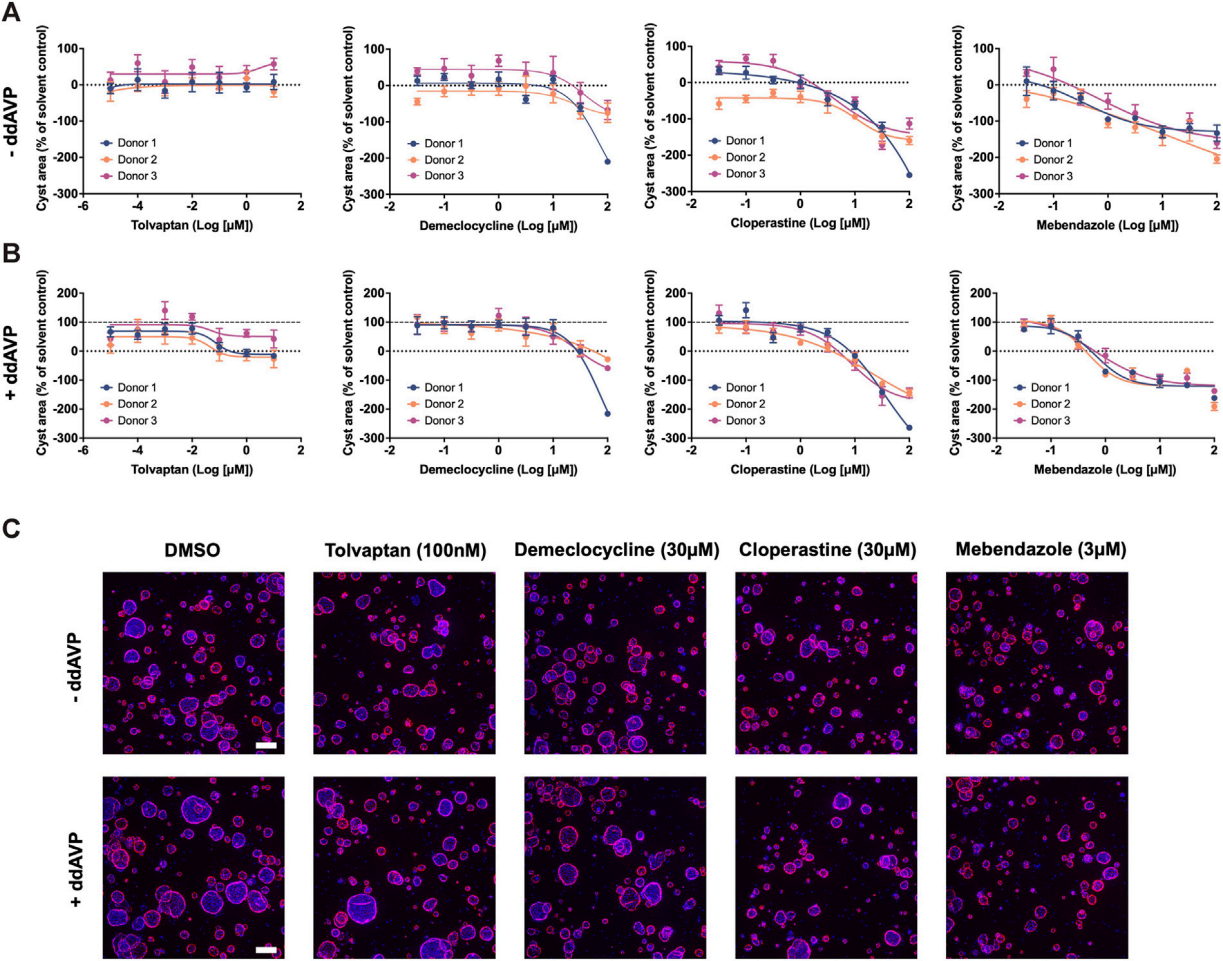
Drugs predicted by computational drug matching inhibit cystic growth in 3D human cellular models of ADPKD. **(A)** In the absence of ddAVP stimulation, tolvaptan has no effect on cyst growth, whereas demeclocycline, cloperastine and mebendazole dose dependently inhibit cyst growth in primary cell cultures derived from three ADPKD donor kidneys. **(B)** Cyst growth is augmented in the presence of ddAVP stimulation, and is dose-dependently inhibited by tolvaptan, demeclocycline, cloperastine and mebendazole in primary cell cultures derived from three ADPKD donor kidneys. Dotted line at 0% represents unstimulated cyst growth, while the dashed line at 100% represents ddAVP-stimulated cyst growth. **(C)** Representative images of 3D-cultured cysts from Donor 2 treated with control and test compounds, with Hoechst staining in blue for nuclei and rhodamine-phalloidin staining in pink for cellular f-actin. Cell cultures were derived from resected kidney tissue of ADPKD patients, and carry the following PKD1 mutations: c.10594C>T Gln3532* (Donor 1), c.5622G>A p.Trp 1874* (Donor 2) and c.5861dup p. (Asn1954Lysfs*36) (Donor 3). Error bars represent standard deviation. Scale bars in **(C)** represent 200 µm.

In order to uncover new therapeutic avenues for ADPKD, we made use of two independent but complementary drug prediction paradigms. The first, disease gene expression mapping (DGEM), is based on connectivity mapping (Lamb et al., 2006), and predicated on the concept that disease-induced perturbations in gene expression can be used to query libraries of drug-induced gene expression signatures in order to identify drugs which might induce therapeutic transcriptional changes in a given disease state. In order to implement this approach for ADPKD, we created disease signatures from publicly available datasets which represented early and late stage disease states, as well as disease progression, in both mouse models of disease and human patient tissue (Song et al., 2009; Pandey et al., 2011; Menezes et al., 2016). We then used these signatures to query the CMap drug database in order to identify drug signatures with the highest connectivity to disease states. While variations of this approach have proven successful in identifying new therapeutic candidates across numerous diseases (Musa et al., 2017), there are a number of limitations, including the limited size of the CMap library, and the exclusive use of human cancer cell lines for deriving its drug perturbation signatures. For these reasons, we also made use of an ADPKD-augmented knowledge graph, from which novel drug-ADPKD links were derived by a neural network trained to recognise graph characteristics of known treatments for similar diseases, via an algorithm termed degree-weighted path count (DWPC). We combined the output from the DGEM and DWPC predictive modules, and prioritised thirteen drugs for preclinical evaluation based on the strength of each prediction across computational prediction sets, novelty, as well as clinical and regulatory considerations which might enable rapid clinical translation. A full list of these prioritised predictions can be seen in Supplementary Table S1. In order to evaluate these drug predictions, we made use of a patient-derived *in vitro* model system which recapitulates three dimensional (3D) cyst growth in the correct genetic context, and has previously been used to interrogate novel therapeutic strategies (Dagorn et al., 2023). We evaluated drug predictions alongside tolvaptan in the presence and absence of the cyst swelling stimulus desmopressin (ddAVP), and determined that three predicted drugs—demeclocycline, cloperastine and mebendazole—demonstrated dose-dependent inhibition of cyst growth in both ddAVP-stimulated and unstimulated conditions, while tolvaptan only exhibited dose-dependent effects on cyst growth in the presence of ddAVP, as expected based on its mechanism of action (Reif et al., 2011) (Figure 1). We next used information and data generated during the prediction process to gain insights into the potential therapeutic mechanisms of these active molecules.

**FIGURE 2:**
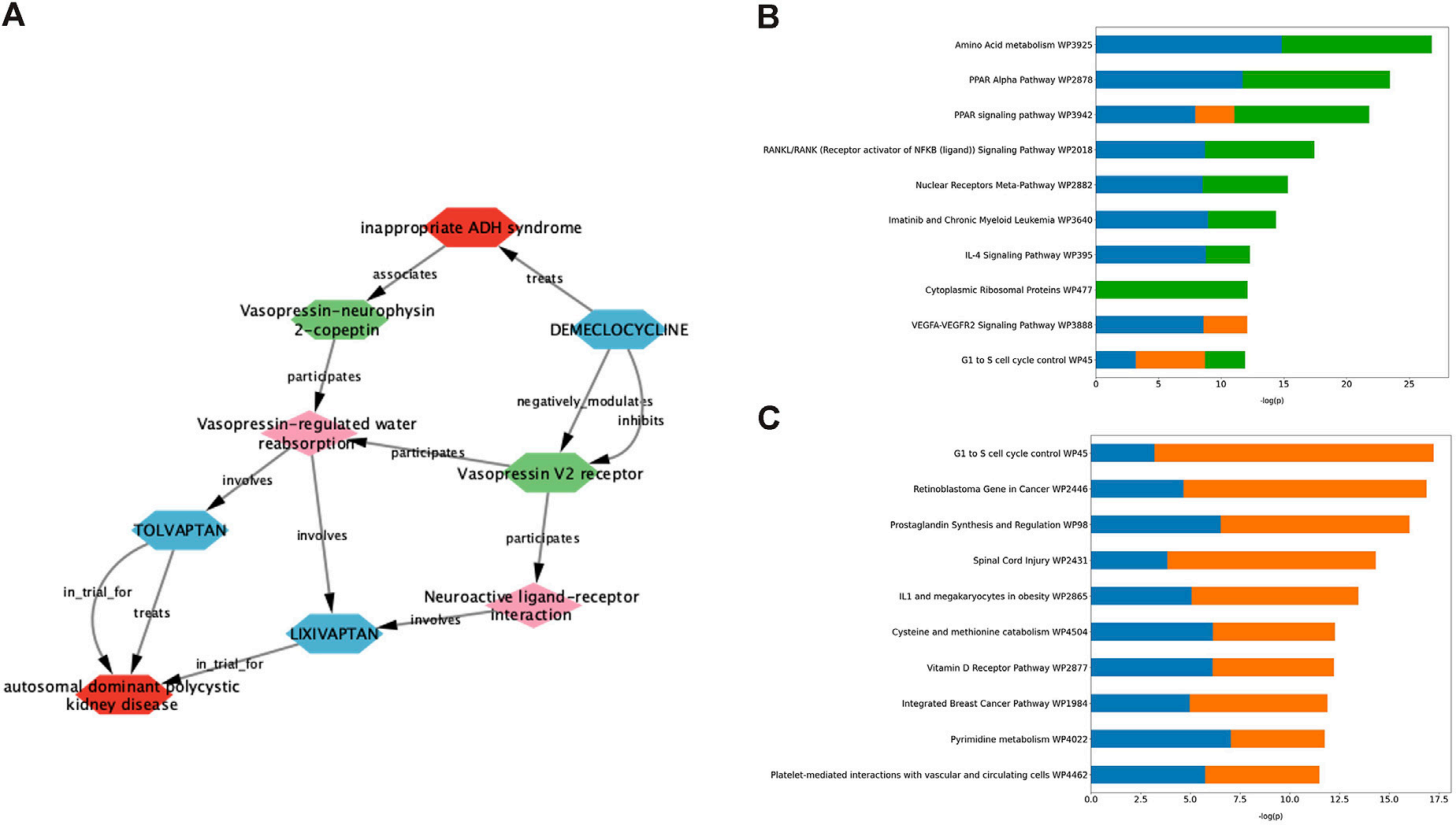
Exploration of data underlying predictive modules provides insight into pathways potentially involved in the therapeutic effect of validated drug predictions. **(A)** Deeper interrogation of the ADPKD-enriched knowledge graph, which gave rise to demeclocycline as a drug prediction, suggests that vasopressin signalling is the causal link between demeclocycline and ADPKD. **(B, C)** Consensus gene enrichment analysis of the gene expression overlap between ADPKD disease signatures (as outlined in Table 1) and cloperastine **(B)** and mebendazole **(C)** drug signatures suggests biological pathways (WikiPathways 2019) which may be relevant in describing the therapeutic effect in each case. In **(B)**, ADPKD-cloperastine gene expression enrichment is represented separately for ADPKD disease signatures 175 (blue), 203343 (orange) and 176 (green). In **(C)**, ADPKD-mebendazole gene expression enrichment is represented separately for ADPKD disease signatures 203348 (blue) and 203349 (orange).

Demeclocycline is a tetracycline antibiotic which was first described over half a century ago. It was predicted as a potential treatment for ADPKD via interrogation of an ADPKD-enriched knowledge graph, and upon further investigation, it appeared this prediction was driven primarily via links to vasopressin signalling (Figure 2A). Indeed, aside from its use in the treatment of susceptible bacterial infections, demeclocycline has been employed for several decades in the treatment of inappropriate antidiuretic hormone syndrome; a feature shared with the standard of care in ADPKD, tolvaptan. In this regard, demeclocycline acts as a physiological antagonist of the V2R, reducing the abundance of adenylate cyclases downstream of V2R, and subsequently leading to a reduction in cAMP-dependent aquaporin 2 transcription (Kortenoeven et al., 2013). The intersection of this mechanism with the direct effects of tolvaptan on the V2R, and the known involvement of this pathway in ADPKD pathogenesis have led some to speculate that demeclocycline may indeed be an efficacious treatment (van Hastel and Torres, 2017), though until now definitive evidence of this hypothesis has been lacking.

The antitussive agent cloperastine has several known targets, including histamine, as well as the sigma receptors σ1 and σ2 which are thought to be responsible for its primary antitussive effect (Gregori-Puigjané et al., 2012). In the current study, cloperastine was predicted using transcriptomic drug matching, thus we used gene enrichment analysis to interrogate the gene expression overlap between drug signatures and disease signatures with the highest connectivity scores, in order to explore pathways which might be relevant for its treatment effect in ADPKD. The highest ranked consensus pathways for the cloperastine-ADPKD gene expression overlap were associated with amino acid metabolism, peroxisome proliferator-activator receptor (PPAR) signalling, nuclear factor κB (NF-κB) and nuclear receptors (Figure 2B). These pathways have been linked to varying degrees to the disease biology of ADPKD (Harris and Torres, 2014), however the link to the primary pharmacology of cloperastine was initially less obvious. Interestingly, a molecular target recently associated with cloperastine is a methyltransferase, protein arginine methyltransferase 5 (PRMT5) (Prabhu et al., 2023). PRMT5 regulates gene expression via the dimethylation of arginine residues in histone and non-histone protein targets, and has been associated with the direct control of both NF-κB (Wei et al., 2013) and PPAR (Huang et al., 2018) transcriptional regulation, which align with the top consensus pathways identified here in the cloperastine-ADPKD gene expression overlaps. Furthermore, PRMT5 has been associated with the regulation of cell cycle and immune cell invasion in the context of cancer (Gu et al., 2012; Abe et al., 2023), which intersect with the lower ranked cloperastine-ADPKD pathways described here. Whether PRMT5 represents a new therapeutic target for ADPKD remains to be determined, however there are a number of clinical candidates evaluating the potential of this target for oncology indications (Zheng et al., 2023). Unfortunately, in addition to many potentially therapeutic targets, cloperastine is also a potent inhibitor of human ether-a-go-go (hERG) and induces QT prolongation *in vivo*, thus demonstrating undesirable proarrhythmic potential (Takahara et al., 2012). While there are obvious limitations in the repurposing of antibiotics or proarrhythmic drugs for a chronic indication such as ADPKD, the ability of our methods to predict demeclocycline and cloperastine as active agents in line with known pharmacology in the case of demeclocycline, and potentially new pharmacology in the case of cloperastine validates the potential of our *in silico* methodology to identify known and novel treatment approaches.

Mebendazole is a broad spectrum anthelmintic agent that has been in clinical and veterinary use for over 50 years. It is believed to mediate its therapeutic effect via binding to parasitic β-tubulin and subsequent inhibition of microtubule polymerisation, thus disrupting key cellular processes leading to paralysis and death of the parasite (Lacey, 1988). In addition to its affinity for parasitic β-tubulin, mebendazole also inhibits microtubule polymerisation in mammalian cells, and moreover demonstrates affinity for a number of kinase targets (Nygren et al., 2013; Issa et al., 2015). These properties, combined with an extensive history of safe human use, have led to significant interest and advancements in the repurposing of mebendazole for oncological indications (Sasaki et al., 2002; Bai RY. et al., 2015; Choi et al., 2021; Gallia et al., 2021; Mansoori et al., 2021; Williamson et al., 2021; Hegazy et al., 2022). Like cloperastine, mebendazole was also predicted as a putative treatment for ADPKD using transcriptomic drug matching methods, and so we again performed gene enrichment analysis of the drug-disease gene expression overlap using the most highly connected disease contrasts in order to gain insight into the potential mechanisms underlying the therapeutic effect in this case (Figure 2C). Key pathways identified from this gene expression overlap included those related to cell cycle, cancer, inflammation and metabolism. These pathways are all highly relevant for both ADPKD and cancer, which are known to share many molecular similarities (Seeger-Nukpezah et al., 2015), and suggest that mebendazole may have a similar mode of action in the treatment of both disease entities. Due to the potent effects of mebendazole on human cellular models of ADPKD, and its previously demonstrated potential as a clinical repurposing candidate in oncology, we decided to focus further efforts on *in vivo* validation and mechanism-based studies to understand if mebendazole and/or its targets might also represent a therapeutic strategy for ADPKD.

**FIGURE 3:**
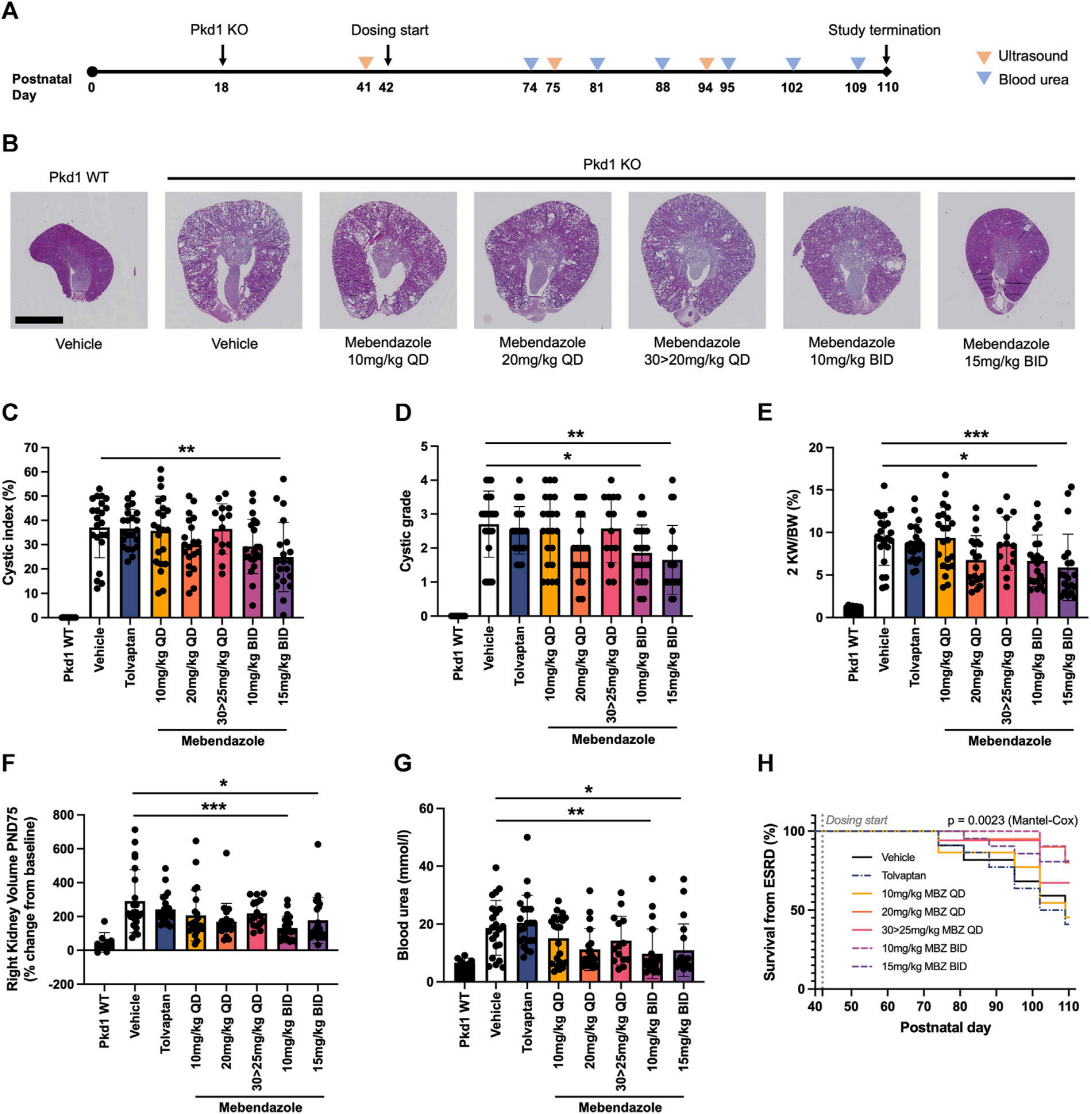
Mebendazole ameliorates kidney phenotypes in the P18 iKsp-*Pkd1* ^del^ mouse model of ADPKD. **(A)** Pkd1 disruption was induced by daily tamoxifen administration from PND18-20, and mebendazole, tamoxifen or vehicle were administered from PND42-110. Ultrasound was used to measure kidney volume at PND41 (baseline), PND75 and PND94, while blood urea was measured weekly from PND74 and used to determine when animals reached ESRD (blood urea >20 mmol/L). **(B)** As demonstrated in hematoxylin and eosin stained kidney sections from PND110 animals, Pkd1 disruption results in enlarged cystic kidneys, which are attenuated with twice daily mebendazole treatment (scale bar represents 2.5 mm). **(C)** Twice daily treatment with 15 mg/kg mebendazole significantly reduces cystic index of Pkd1 KO mice; ** = *p* < 0.01 Dunnet’s multiple comparisons test. **(D)** Twice daily treatment with 10 mg/kg or 15 mg/kg mebendazole significantly reduces cystic grade in Pkd1 KO mice, as assessed by blinded histopathological scoring; * = *p* < 0.05 and ** = *p* < 0.01 Dunn’s multiple comparisons test. **(E)** Body weight normalised kidney weight is increased in Pkd1 KO mice, but significantly attenuated with twice daily treatment with 10 mg/kg or 15 mg/kg mebendazole; * = *p* < 0.05 and *** = *p* < 0.001 Dunnet’s multiple comparisons test. **(F)** Twice daily treatment with 10 mg/kg or 15 mg/kg mebendazole significantly reduces increased kidney volume in Pkd1 KO mice at the study dosing mid-point PND75; * = *p* < 0.05 and *** = *p* < 0.001 Dunn’s multiple comparisons test. **(G)** Terminal blood urea is elevated in Pkd1 KO mice, but attenuated by BID treatment with 10 mg/kg or 15 mg/kg mebendazole; * = *p* < 0.05 and ** = *p* < 0.01 Dunn’s multiple comparisons test. **(H)** Kaplan-Meier analysis of kidney survival from ESRD reveals a significant treatment effect. **(C–G)** Each symbol represents one animal; error bars represent standard deviation; *n* = 14–22 per treatment group (Pkd1 WT group excluded from analysis).

In order to assess the efficacy of mebendazole against ADPKD-relevant phenotypes *in vivo*, we utilised the P18 iKsp-*Pkd1* ^del^ mouse model, which employs a tamoxifen-inducible cadherin promoter to selectively disrupt Pkd1 expression in the kidney epithelium at postnatal day (PND) 18, and has previously been employed to evaluate new therapeutic strategies (Dagorn et al., 2023; Song et al., 2023). While perinatal inactivation of Pkd1 in this genetic background produces a rapid and severe model of kidney cystogenesis and functional decline over a compressed timeline, inactivation of Pkd1 at PND18 induces an adult-onset, slowly progressing model of cystic kidney disease which leads to renal failure over the course of several months, and offers the opportunity to evaluate treatment strategies over a chronic time window (Leeuwen et al., 2007; Leonhard et al., 2016). After disruption of the Pkd1 locus at PND18, mice were treated from PND42 with once daily (QD; 10, 20 or 30 mg/kg) or twice daily (BID; 10 or 15 mg/kg) mebendazole via oral gavage, or tolvaptan dosed in medicated food (0.1% w/w) until study termination (Figure 3A). The study was terminated at PND110, 1 day after >50% of animals in the untreated control group had been euthanised due to end-stage renal disease (ESRD), defined as a blood urea level >20 mmol/L. Weekly body weight measurements indicated that mebendazole doses of 10 mg/kg QD, 20 mg/kg QD, 10 mg/kg BID and 15 mg/kg BID were well tolerated in these mice, however some animals treated with 30 mg/kg QD experienced weight loss, which necessitated a dosing holiday and reduced dose of 25 mg/kg QD in this group from PND59 (Supplementary Figures S1A, B). Despite this intervention, only 14/22 animals made it to the study endpoint or termination in this treatment group, compared with almost all animals from the remaining treatment groups, suggesting the maximum tolerated exposure of mebendazole was exceeded in this group (Supplementary Figure S1C). Over the course of this study, P18 iKsp-*Pkd1* ^del^ mice developed highly cystic kidneys which were on average 7-fold the normalised weight of Pkd1 wildtype mouse kidneys (Figures 3B–E). Twice daily oral administration of mebendazole at 15 mg/kg significantly alleviated kidney cystic load by 33% (Figure 3C), while twice daily administration of mebendazole at 10 mg/kg and 15 mg/kg, significantly reduced kidney cystic grade as assessed by histopathological scoring (Figure 3D). In addition to effects on cystic load, twice daily administration of mebendazole at 10 mg/kg and 15 mg/kg also significantly ameliorated increased kidney weight by 32% and 42%, respectively (Figure 3E). As a further measure of structural disease progression, we took advantage of the chronic nature of this model system to evaluate kidney volume longitudinally using a non-invasive ultrasound imaging approach. On PND75, kidney volume in Pkd1 wildtype mice was increased by an average of 47% compared with pre-dosing values taken on PND41, whereas in the same timeframe, kidney volume in vehicle treated Pkd1 KO mice increased by an average of 291%, consistent with the development of enlarged cystic kidneys by the midpoint in this study (Figure 3F). Consistent with ameliorative effects on terminal kidney structure, twice daily administration of 10 mg/kg and 15 mg/kg mebendazole significantly reduced kidney volume expansion at this point in the study compared with vehicle treated Pkd1 KO mice, from an average of 291% to 132% and 177%, respectively (Figure 3F). By PND94, only twice daily administration of 15 mg/kg had a significant effect on reducing kidney volume in Pkd1 KO mice, however it should be noted that by this late stage of the study, animals in the comparator vehicle-treated Pkd1 KO group had begun to enter ESRD and drop out of the study, potentially reducing statistical power to detect differences in kidney volume between treatment groups (Supplementary Figure S2). In order to evaluate the effects of mebendazole on preserving kidney function, we compared blood urea levels in each animal from terminal samples taken when animals reached ESRD, or at the study terminus, whichever came earlier. Terminal blood urea levels were an average of 6.7 mmol/L in Pkd1 WT mice, and 18.7 mmo/L in vehicle treated Pkd1 KO mice, indicating substantial functional decline across the course of the study (Figure 3G). Twice daily treatment with 10 mg/kg and 15 mg/kg mebendazole significantly reduced blood urea levels by 48% and 41% to 9.8 mmol/L and 11.0 mmol/L, respectively, compared with vehicle treated Pkd1 KO mice (Figure 3G), suggesting a significant rescue of renal functional decline in mebendazole-treated Pkd1 KO mice, while kidney survival analysis further confirmed a significant treatment effect (Mantel-Cox test *p* < 0.01) (Figure 3H).

These findings demonstrate that mebendazole treatment ameliorates ADPKD phenotypes in a chronic, slowly-progressing mouse model of disease, and further suggest that increasing the frequency of mebendazole administration to deliver the same dose over two administrations in order to maintain constant levels of exposure improves both efficacy and tolerability. In contrast to mebendazole, the positive control and current standard of care for ADPKD patients, tolvaptan, surprisingly failed to produce significant effects on any measured endpoints. It should be noted that constant exposure to therapeutic levels of tolvaptan is required, which can be difficult to control by using the standard approach of tolvaptan dosing via medicated food. Based on the significant effects of mebendazole on kidney cyst growth in human cellular models and an *in vivo* model of ADPKD, we next focused on determining the likely mechanism behind this therapeutic effect.

Given the multifactorial nature of ADPKD pathophysiology, and the polypharmacological nature of mebendazole, we made use of a recently described computational framework called the multiscale interactome to understand how mebendazole may treat ADPKD at the molecular level (Ruiz et al., 2021). This framework is predicated on the concept that drugs often do not treat diseases via direct modification of disease-associated proteins, but via a complex propagation of signals through protein-protein interactions and biological functions and processes. When these complex drug-disease relationships are represented as diffusion profiles, they can be used to understand how a given drug might impact a given disease, particularly in cases where a direct molecular link is not immediately obvious.

**FIGURE 4:**
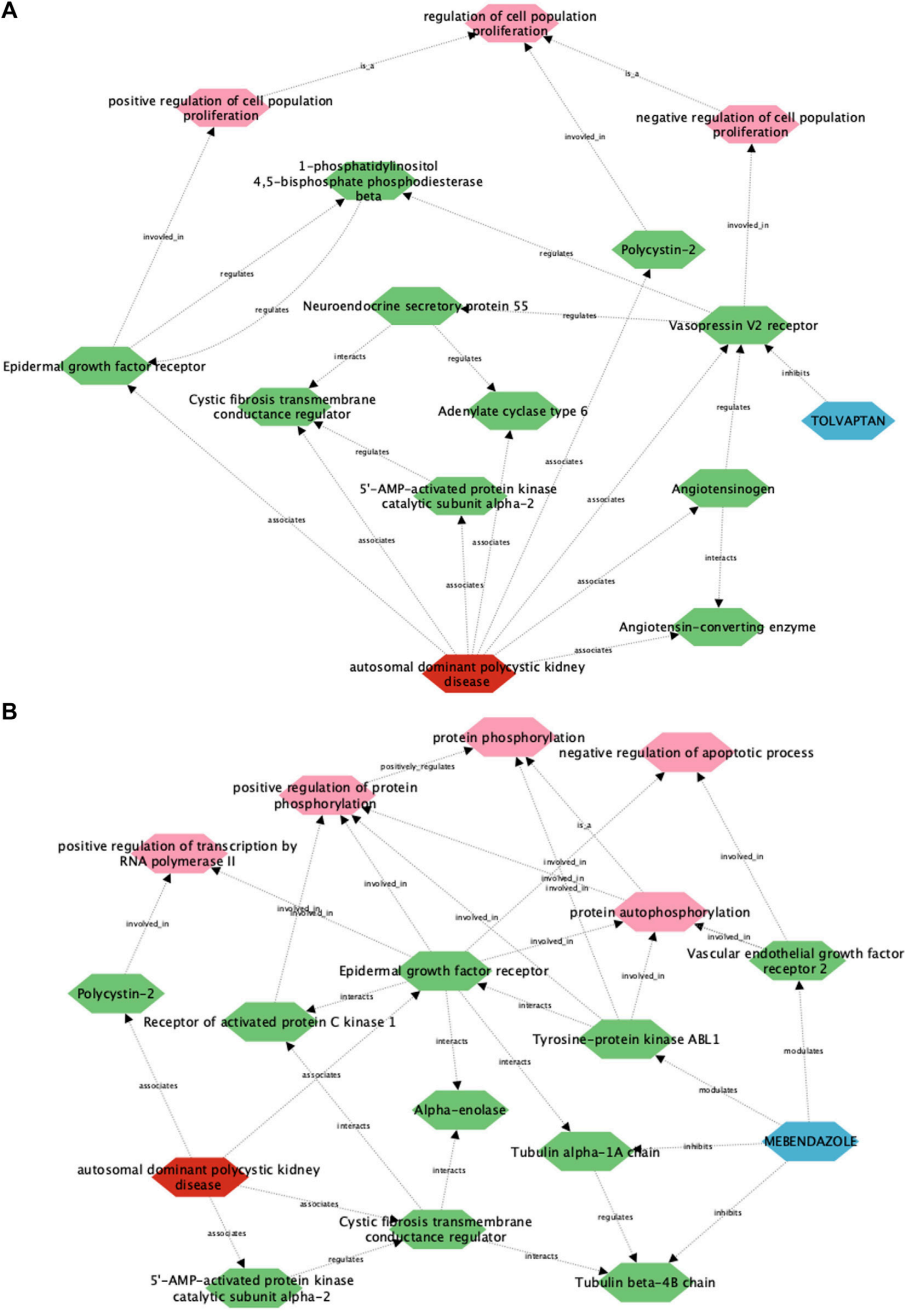
A multiscale interactome approach identifies key proteins and signalling pathways linking the approved treatment, tolvaptan, and the putative treatment, mebendazole, with ADPKD. Using this approach, a link is described between ADPKD disease biology and the approved drug tolvaptan **(A)**, as well as the putative treatment mebendazole **(B)**, in the first case identifying well established drug targets and processes associated with tolvaptan, and in the second case putative targets and processes of mebendazole for further experimental validation.

To understand how this framework performs in describing drug-disease relationships for ADPKD, we initially focused on the well understood link between tolvaptan and ADPKD (Figure 4A). In line with known drug pharmacology and disease biology, the tolvaptan-ADPKD diffusion profile identifies V2R as the sole drug target for tolvaptan, and also identifies key proteins known to be perturbed in the ADPKD disease state, including V2R itself, as well as polycystin-2, angiotensinogen, cystic fibrosis transmembrane conductance regulator (CFTR) and epidermal growth factor (EGFR). While V2R, the molecular target of tolvaptan, is directly linked to PKD pathophysiology (Wang et al., 2008), the diffusion profile also describes the interaction of V2R with other first order ADPKD-associated proteins via additional entities or biological functions, adding more nuance to the drug-treatment picture. There are interactions described in the diffusion profile between V2R and the process of cell proliferation, which is itself dysregulated in ADPKD by the polycystins (Grimm et al., 2006), the renin-angiotensin-aldosterone system, dysfunction of which is both a downstream consequence of, and direct contributor to ADPKD (Hian et al., 2016), and components of the G protein-coupled receptor signalosome, which regulate ADPKD-associated proteins such as CFTR and EGFR (Hama and Park, 2016; Sussman et al., 2020). These findings demonstrate the utility of the multiscale interactome approach in describing complex drug-disease relationships between two well described entities.

We next utilised the same approach to gain insight into the unknown drug-disease relationship between mebendazole and ADPKD (Figure 4B). As anticipated, disease-associated proteins in the mebendazole-ADPKD diffusion profile share significant overlap with those identified in the tolvaptan-ADPKD diffusion profile described above, however the drug targets of mebendazole and their downstream associations are markedly different to those of tolvaptan. Mebendazole is a well known microtubule depolymerising agent, and in line with this mode of action, two tubulin proteins are herein described as drug targets, Tubulin beta 4B, and Tubulin alpha 1A. The diffusion profile makes a direct connection between these microtubule proteins and the ADPKD-associated proteins EGFR and CFTR, trafficking of which has indeed previously been linked to the state of the microtubule network (Morris et al., 1998; Liu et al., 2012), and may explain in part the downstream effects of mebendazole-induced microtubule destabilisation on ADPKD-relevant networks. Aside from its microtubule disrupting action, mebendazole is also an inhibitor of a number of kinases, and accordingly is linked in the diffusion profile via two of its known protein kinase targets, vascular endothelial growth factor receptor 2 (VEGFR2) (Dakshanamurthy et al., 2012) and ABL1 (aka Abl) (Nygren et al., 2013), to phosphorylation processes also regulated by EGFR. VEGF signalling has previously been linked with ADPKD pathophysiology (Tao et al., 2007), and herein the diffusion profile suggests this could be mediated in part via the regulation of apoptosis, which is itself a key process known to be dysregulated in the disease state (Zhou and Li, 2015). On the other hand, Abl has not been explicitly linked to ADPKD previously, although intriguingly it is linked both here in the diffusion profile and experimentally elsewhere to direct regulation of EGFR (Tanos and Pendergast, 2006). Using a multiscale interactome approach we have identified a number of potential molecular targets and functions that may drive the therapeutic efficacy of mebendazole in ADPKD, which we next sought to explore using experimental approaches.

**FIGURE 5:**
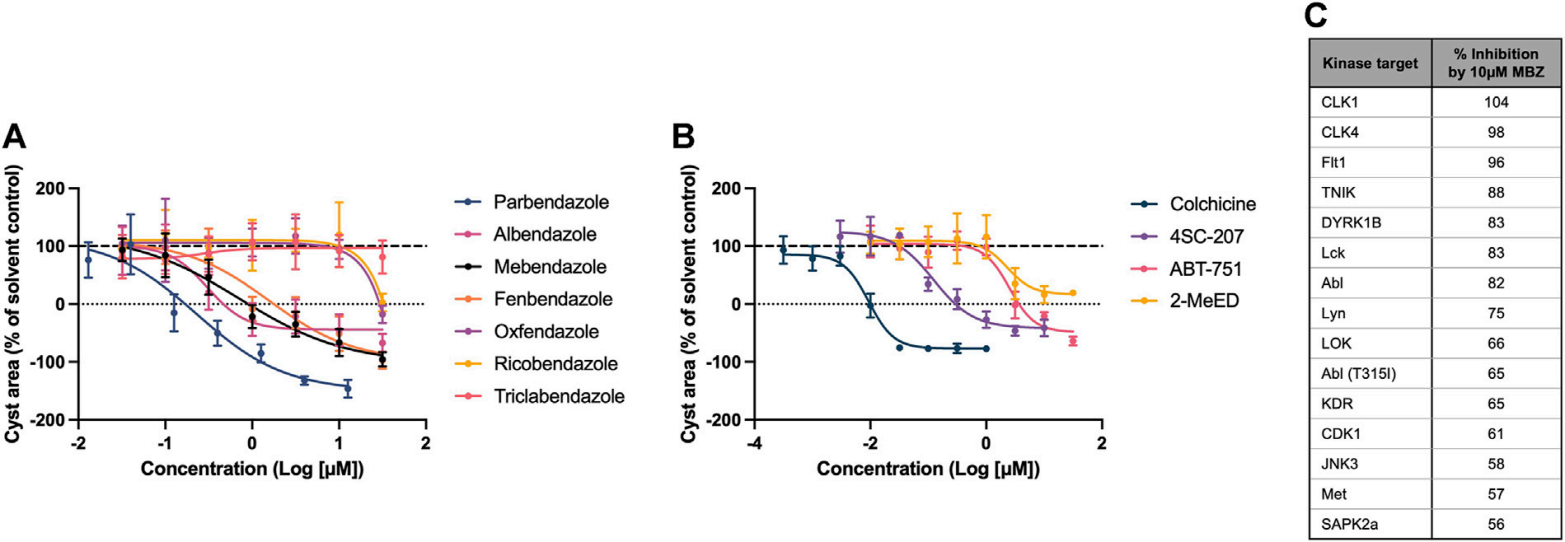
The therapeutic effect of mebendazole in ADPKD is likely driven by an inhibitory effect on microtubule polymerisation and ADPKD-relevant kinases. **(A)** In addition to mebendazole, other benzimidazole anthelmintics with microtubule depolymerising activity are also active in primary cell cultures derived from an ADPKD donor kidney. **(B)** Members of the wider class of CBS ligands with microtubule depolymerising activity are also active in primary cell cultures derived from an ADPKD donor kidney. **(C)** At 10µM, mebendazole (MBZ) inhibits a number of protein kinases, including several ADPKD-relevant kinase targets. Cell cultures in **(A, B)** were derived from resected kidney tissue of an ADPKD patient carrying a **(C)** 5622G>A p.Trp 1874* mutation in PKD1. Error bars represent standard deviation.

Mebendazole is part of the benzimidazole class of anthelmintics, which itself is a member of a wider family of structurally diverse compounds which bind to the colchicine-binding site (CBS) of β tubulin, inhibiting the polymerisation of microtubules. Given its known pharmacology as a microtubule inhibitor, and its predicted link to ADPKD pathophysiology via tubulin protein targets uncovered by the multiscale interactome approach, we evaluated the potency and efficacy of a range of compounds with affinity for the CBS in the 3D phenotypic assay, to establish if the therapeutic effect of mebendazole in ADPKD is indeed being driven primarily via this mechanism. We determined that in addition to mebendazole, a number of benzimidazole anthelmintics (Figure 5A) and CBS agents from the wider family (Figure 5B) demonstrate dose-dependent inhibition of kidney cyst growth. Given the additional link of mebendazole to ADPKD via two protein kinase targets predicted by the multiscale interactome approach, we assessed the ability of mebendazole to inhibit a diverse panel of kinases, which was enriched with additional kinase targets previously known or predicted to be inhibited by mebendazole, or previously implicated in ADPKD. Utilising a cell-free assay platform, we observed that mebendazole inhibited 15 out of 94 tested kinases by >50% at 10 µM (Figure 5C). Both of the kinase targets predicted in the mebendazole-ADPKD diffusion profile, VEGFR2 (KDR) and Abl, were inhibited by mebendazole at this concentration, while overall four targets with known links to ADPKD pathophysiology were represented: VEGFR1 (Flt1) and VEGFR2 (KDR) (Bello-Reuss et al., 2001; Tao et al., 2007), cyclin dependent kinase 1 (CDK1) (Zhang et al., 2021) and Met (Qin et al., 2010). While the consistent activity of a wide range of microtubule polymerisation inhibitors on cyst growth *in vitro*, which in the case of the benzimidazole anthelmintics broadly correlates with their biochemical potency in inhibiting mammalian tubulin polymerisation (Lacey, 1988), and includes molecules such as albendazole with no appreciable kinase activity (Nygren et al., 2013), suggests that mebendazole acts primarily via microtubule polymerisation inhibition to inhibit cyst growth in ADPKD, there remains an intriguing possibility that inhibition of several protein kinase targets also contributes to the therapeutic effect of mebendazole observed in preclinical ADPKD models. In summary, *in silico* modelling and pharmacological and biochemical data support the notion that the primary effects of mebendazole in ADPKD are likely driven via binding to the CBS of β tubulin, inhibition of microtubule polymerisation, and disruption of downstream microtubule dynamics, with a possible contribution via the inhibition of select protein kinase targets.

## Discussion

Using transcriptomic and machine-learning drug discovery approaches, we predicted a number of existing drugs which may have therapeutic potential in ADPKD, and subsequently validated several of these predictions in relevant disease model systems. Among these predictions, the anthelmintic drug and anti-cancer repurposing candidate mebendazole ameliorated cyst growth in multiple human cellular models of ADPKD, and disease-relevant phenotypes in a slowly progressing mouse model of ADPKD, inhibiting both kidney cyst growth and kidney size expansion, while rescuing declining kidney function. *In silico* insight and mechanistic studies revealed that the anti-cystic effect of mebendazole in ADPKD is likely driven primarily by its inhibitory effect on microtubule polymerisation, with a potential contribution from the inhibition of protein kinase targets known or predicted to be involved in disease pathophysiology.

In addition to mebendazole, we determined that a number of well known microtubule depolymerising agents attenuated cystic growth in a human cellular model of ADPKD. Aside from a fundamental role in cell division, microtubules also form the core axoneme structure of the cilia, and facilitate essential functions such as cilia assembly/disassembly, and intraflagellar transport of ciliary proteins (Conkar and Firat-Karalar, 2021). Previous research has highlighted the importance of the microtubule cytoskeleton in kidney cyst formation and progression, and the impact of targeting microtubule dynamics and function on disease pathophysiology. As early as 1994, researchers using paclitaxel observed that hyperstabilisation of microtubules was sufficient to blunt kidney cyst expansion *in vitro* and *in vivo* (Woo et al., 1994), and more recently it was shown that the microtubule stabilising compound 1-Indanone was able to rescue ADPKD phenotypes by correcting abnormal cilia length and aberrant cilia signalling pathways (Li et al., 2023). A recent phenotypic screening effort identified a number of microtubule stabilisers as well as destabilisers as anti-cystic agents in murine and human cellular models of ADPKD, suggesting that depolymerisation as well as hyperstabilisation might both be viable therapeutic strategies (Asawa et al., 2020). The diverse functions of the microtubule network are facilitated in part through extensive posttranslational modifications of its α and β tubulin subunits (Wloga et al., 2017), and recent findings have highlighted the potential of targeting these modifications in polycystic kidney disease. Acetylation of α-tubulin is associated with stable microtubule polymers, and is primarily regulated by the opposing actions of the acetylase α-tubulin acetyltransferase 1, and the deacetylases histone deacetylase 6 (HDAC6) and sirtuin 2 (SIRT2) (Li and Yang, 2015). Loss of polycystin-1 is associated with an increase in HDAC6 (Liu et al., 2012) and SIRT2 (Zhou et al., 2014) expression, and a decrease in acetylated α-tubulin (Zhou et al., 2014), while inhibition of either deacetylase has been shown to augment α-tubulin acetylation levels and ameliorate ADPKD-relevant phenotypes (Zhou et al., 2014; Cebotaru et al., 2016; Yanda et al., 2017). While non-selective targeting of microtubule dynamics has the potential to induce undesirable toxicity due to the role of microtubules in essential cellular processes (Dumontet and Jordan, 2010), selective targeting of dysfunctional elements of the network such as this might open up the possibility of more tractable treatment opportunities for ADPKD. Indeed, while targeting HDAC6 has shown promise in preclinical models of ADPKD as described above, HDAC6-null mice with hyperacetylated α-tubulin are viable and develop normally (Zhang et al., 2008), suggesting that disease-selective targeting of the microtubule network might be possible to achieve while limiting undesirable toxicity.

Numerous growth factors and their respective protein kinase-mediated signalling pathways have been implicated in ADPKD, including among others epidermal growth factor, insulin growth factor, platelet-derived growth factor and vascular endothelial growth factor (VEGF) (Formica and Peters, 2020). While interventions targeting a number of these pathways have demonstrated promise in preclinical models of polycystic kidney disease, only the Src/Bcr-Abl inhibitor bosutinib and the multi-kinase inhibitor tesevatinib have thus far progressed into randomised clinical trials (Zhou and Torres, 2023). Using the multiscale interactome framework, we predicted that in addition to tubulin, mebendazole may also be acting via kinase inhibition to elicit a treatment response in ADPKD. To explore this prediction experimentally, we screened mebendazole against a kinase panel enriched with targets predicted or known to be inhibited by mebendazole, or important in ADPKD, and determined that in addition to the predictions VEGFR2 and Abl, mebendazole also inhibited VEGFR1, Met and CDK1, which have all been implicated in ADPKD. These findings corroborate existing literature - which is drawn upon by the multiscale interactome method to make inferences - by replicating direct inhibition of VEGF2 and Abl by mebendazole (Dakshanamurthy et al., 2012; Issa et al., 2015; Ariey-Bonnet et al., 2020), and also extend these studies by implicating mebendazole in the inhibition of additional ADPKD-relevant kinase targets it has not previously been demonstrated to interact with. A number of studies have linked pro-angiogenic VEGF signalling to ADPKD progression, although the picture is admittedly complex: suppression of either VEGFR1 or VEGFR2 expression suppressed cyst growth in the Han:SPRD model of polycystic kidney disease (Tao et al., 2007), but paradoxically anti-VEGF treatment augmented cyst growth and accelerated functional decline in the same model (Raina et al., 2011). Almost 30 years ago, it was observed that renal-cyst lining cells of PKD patients aberrantly expressed both the mitogenic hepatocyte growth factor and its receptor tyrosine kinase Met (Horie et al., 1994), while further mechanistic studies confirmed hyperactivation of Met signalling in Pkd1 models, pharmacological inhibition of which rescued disease phenotypes (Qin et al., 2010). Finally, a recent study identified the cell cycle regulator CDK1 as a key promoter of early cystogenesis in ADPKD, and crucially demonstrated that genetic ablation of this gene inhibited cell cycle progression and ameliorated kidney disease phenotypes *in vivo* (Zhang et al., 2021). Whether interactions with any or all of these targets and signalling pathways in addition to the microtubule network is essential for the activity of mebendazole in ADPKD requires further experimental validation, however a synergistic effect of mebendazole on tubulin and protein kinases has been hypothesised to underlie its potent anticancer activity (Nygren et al., 2013; Bai R.-Y. et al., 2015), and may make biological sense in the current context given the similarities in signalling pathways between ADPKD and cancer (Seeger-Nukpezah et al., 2015), and the representation of cancer-related pathways in the mebendazole-ADPKD gene expression overlap. A polypharmacological mode of action may indeed be desirable in a multifaceted disease such as ADPKD.

This study builds on previous work using computational methods to identify new drugs and therapeutic candidates for ADPKD. Malas and colleagues utilised a combined transcriptomic and cheminformatic approach to prioritise and ultimately validate several novel compounds in a 3D cystic assay (Malas et al., 2020). Their approach involved the creation of a disease-stage specific transcriptomic signature, from which candidate genes were identified and linked to molecules through publicly available drug databases. Targets and molecules were ultimately filtered for validation based on biological and chemical insights, and the potential for clinical translation. Earlier this year, Wilk et al., 2023 applied a similar transcriptomic approach to us, in that case making use of publicly available transcriptomic datasets to create Pkd2-specific ADPKD disease signatures, from which signature reversion was sought from the Library of Integrated Network-based Cellular Signatures (LINCs) drug signature database in order to identify drug repurposing candidates. While one group has previously made use of a knowledge graph-based approach to prioritise preclinically active compounds with the highest chance of clinical translation (Malas et al., 2019), to our knowledge, the current study provides the first combined application of transcriptomic and machine-learning approaches to identify and prioritise putative treatments for ADPKD, and further deconvolute potential mechanisms of action for experimental validation.

In summary we report, using computational, *in vitro* and *in vivo* approaches, that the anthelmintic drug mebendazole ameliorates disease-relevant phenotypes in cellular and animal models of ADPKD. We further show that this effect is likely primarily due to the inhibitory effect of mebendazole on the polymerisation of microtubules, which underlie cellular processes important in ADPKD, including cell proliferation, transport, and cilia signalling, and extends previous work linking the importance of the microtubule network to ADPKD pathophysiology. We also describe the inhibitory profile of mebendazole on known and novel protein kinase targets, some of which have previously been implicated in ADPKD, suggesting mebendazole may be acting via polypharmacology to impact disease mechanisms. We acknowledge that further experimental efforts will be required to confirm the actions of mebendazole on these putative targets in relevant disease model systems. It would be particularly informative to investigate these mechanisms in dedicated *in vivo* studies, where the effects of mebendazole on a wider range of ADPKD-relevant cell types and phenotypes could be evaluated. Notwithstanding these limitations, this work supports the combined role of *in silico* and experimental approaches in the discovery of new treatments and therapeutic pathways for rare diseases with complex or poorly understood pathophysiology.

## Data Availability

The datasets presented in this study can be found in online repositories. The names of the repository/repositories and accession number(s) can be found in the article/Supplementary Material.

## In Memoriam

Dedicated to the memory of Neil T. Thompson.

## Supplementary material

SUPPLEMENTARY FIGURE S1 Body weight measurements and study dropouts per treatment group in Pkd1 KO mice throughout the P18 iKsp-*Pkd1* ^del^ mouse study. **(A)** Body weight consistently climbed in line with the study vehicle group in the 10 mg/kg QD and 20 mg/kg QD mebendazole groups, however average body weight plateaued in the 30 mg/kg QD group, necessitating a dose reduction to 25 mg/kg QD from PND59. **(B)** Body weight consistently climbed in line with the study vehicle group in the 10 mg/kg BID and 15 mg/kg BID dosing groups. **(C)** Number of dropouts in each treatment group due to spontaneous death or euthanasia not associated with ESRD (blood urea >20 mmol/L). *n* = 22/Pkd1 KO group at start of the study. Error bars represent standard deviation.

SUPPLEMENTARY FIGURE S2 Twice daily mebendazole ameliorates enlarged kidney volume as measured by ultrasound at PND94. Twice daily treatment with 15mg/kg mebendazole significantly reduces increased kidney volume in Pkd1 KO mice at PND94; * = p < 0.05 Dunn’s multiple comparisons test. Error bars represent standard deviation.

SUPPLEMENTARY TABLE S1 Drug predictions prioritised for preclinical evaluation. Predictions were made using DGEM applied to six ADPKD disease contrast gene expression sets, and DWPC exploration of an ADPKD-enriched knowledge graph. Absolute connectivity scores in the case of DGEM-derived predictions, and relative prediction scores in the case of DWPC-derived predictions are represented in colour scale.
