# Supplementary material for: Computational drug discovery approaches identify mebendazole as a candidate treatment for autosomal dominant polycystic kidney disease
Source: Front Pharmacol. 2024 May 23;15:1397864. doi: 10.3389/fphar.2024.1397864 (PMC11154008; doi:10.3389/fphar.2024.1397864)
Supplement: Supplementary file 1 [file Table1.pdf]

		DGEM						DWPC
		GSE24352		GSE72554		GSE7869		
**Drug**	**Concentration (µM)**	203343	203416	203348	203349	175	176	
Chenodiol	10.20							
Cloperastine	11.00							
Cholecalciferol	10.40							
Demeclocycline	8.00							
Homochlorcyclizine	10.40							
Mebendazole	13.60							
Melatonin	17.20							
Perhexiline	10.20							
Prochlorperazine	10.00							
	6.60							
Phenylbutanoic acid	100.00							
	200.00							
	1,000.00							
Sulfasalazine	100.00							
	10.00							
Thioridazine	10.00							
	1.00							
	9.80							
Trifluoperazine	10.00							
	8.40							

	1 (maximum score)
	0 (no prediction)
