# Supplementary figures and images for: Computational drug discovery approaches identify mebendazole as a candidate treatment for autosomal dominant polycystic kidney disease
Source: Front Pharmacol. 2024 May 23;15:1397864. doi: 10.3389/fphar.2024.1397864 (PMC11154008; doi:10.3389/fphar.2024.1397864)

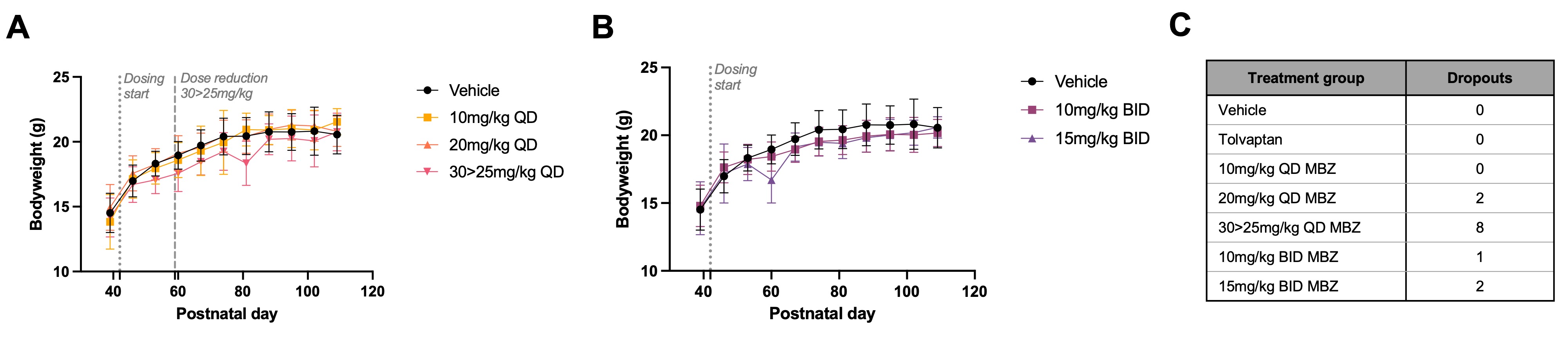

Supplement: Supplementary file 2 [file Image1.JPEG]

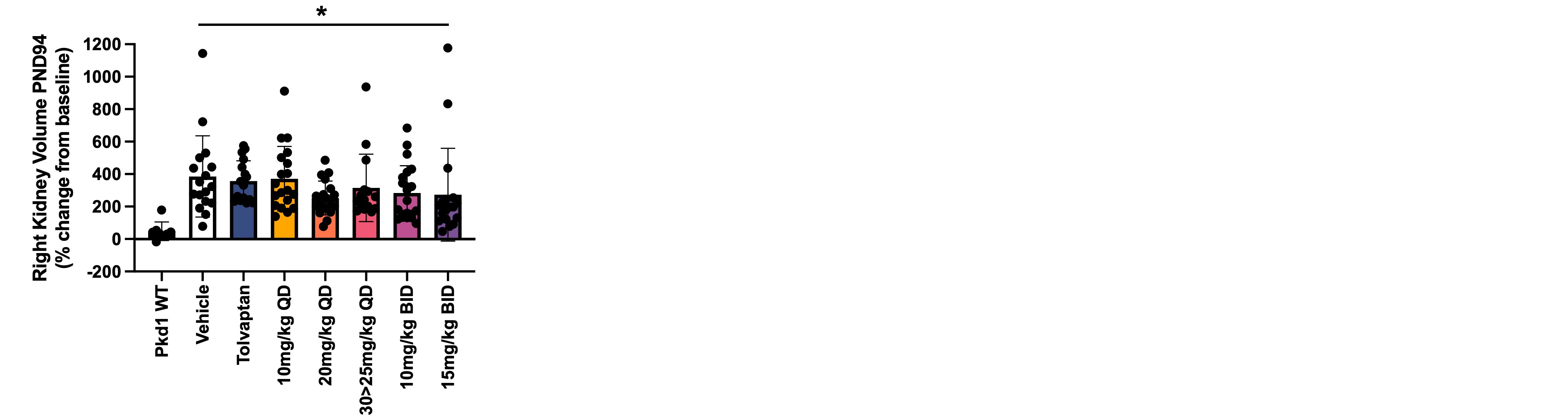

Supplement: Supplementary file 3 [file Image2.JPEG]
